# Frozen Shoulder as a Metabolic and Immune Disorder: Potential Roles of Leptin Resistance, JAK-STAT Dysregulation, and Fibrosis

**DOI:** 10.3390/jcm14051780

**Published:** 2025-03-06

**Authors:** Santiago Navarro-Ledesma

**Affiliations:** Department of Physiotherapy, Faculty of Health Sciences, Campus of Melilla, University of Granada, Querol Street 5, 52004 Melilla, Spain; snl@ugr.es

**Keywords:** frozen shoulder, metabolism, immunology, psychology, rehabilitation

## Abstract

Frozen shoulder (FS) is a complex and multifactorial condition characterized by persistent inflammation, fibrosis, and metabolic dysregulation. Despite extensive research, the underlying drivers of FS remain poorly understood. Recent findings indicate the coexistence of pro-inflammatory and fibrosis-resolving macrophages within affected tissues, suggesting a dysregulated immune response influenced by metabolic and neuroendocrine factors. This review proposes that leptin resistance, a hallmark of metabolic syndrome and chronic inflammation, may play a central role in FS pathogenesis by impairing macrophage polarization, perpetuating inflammation, and disrupting fibrosis resolution. The JAK-STAT signaling pathway, critically modulated by leptin resistance, may further contribute to immune dysregulation by sustaining inflammatory macrophage activation and interfering with tissue remodeling. Additionally, FS shares pathogenic features with fibrotic diseases driven by TGF-β signaling, mitochondrial dysfunction, and circadian disruption, further linking systemic metabolic dysfunction to localized fibrotic pathology. Beyond immune and metabolic regulation, alterations in gut microbiota, bacterial translocation, and chronic psychosocial stress may further exacerbate systemic inflammation and neuroendocrine imbalances, intensifying JAK-STAT dysregulation and leptin resistance. By examining the intricate interplay between metabolism, immune function, and fibrotic remodeling, this review highlights targeting leptin sensitivity, JAK-STAT modulation, and mitochondrial restoration as novel therapeutic strategies for FS treatment. Future research should explore these interconnections to develop integrative interventions that address both the metabolic and immune dysregulation underlying FS, ultimately improving clinical outcomes.

## 1. Introduction

Frozen shoulder (FS), also known as adhesive capsulitis, is a musculoskeletal condition characterized by progressive pain and significant limitation in the range of motion of the shoulder joint. Affecting approximately 2% to 5% of the general population, FS is particularly prevalent among individuals between the ages of 40 and 60, with a higher incidence observed in women compared to men [1,2,3]. The condition can be classified into primary or idiopathic and secondary forms, with the latter often associated with trauma, surgery, or systemic diseases such as diabetes mellitus. FS not only impacts patients’ quality of life, but also has considerable socioeconomic implications, including high healthcare costs and significant work-related disability. Studies have shown that up to 50% of individuals with FS experience a reduction in their capacity to perform work-related tasks, leading to decreased productivity and increased absenteeism [4]. The long duration of the disease, which can last from 1 to 3 years or more, further exacerbates these socioeconomic burdens, as patients often require prolonged treatment and rehabilitation [5].

The etiology of FS remains largely unclear, though it is generally accepted to be a multifactorial condition involving both mechanical and biochemical components [6]. Systemic diseases such as diabetes mellitus, cardiovascular disease, and metabolic syndrome have been strongly associated with an increased risk of developing FS [7]. These conditions, characterized by chronic low-grade inflammation and insulin resistance, contribute to altered tissue repair mechanisms, leading to fibrosis and thickening of the joint capsule [4]. In particular, metabolic disturbances, including the dysregulation of adipokines, have been proposed as central to the inflammatory processes driving FS [8,9]. One of the key adipokines involved is leptin, a hormone produced by adipose tissue that plays a significant role in both energy metabolism and immune regulation. Elevated leptin levels and leptin resistance may be linked to the chronic inflammation and fibrosis observed in FS, suggesting a possible metabolic pathway contributing to the disease’s progression, and positioning it as a pivotal player in the emerging field of immunometabolism [10]. Leptin receptors are widely expressed throughout the immune system, and their regulatory effects encompass both innate and adaptive immune cells. Leptin is among the adipokines contributing to the chronic inflammatory state associated with obesity, which predisposes individuals not only to type 2 diabetes, metabolic syndrome, and cardiovascular disease, but also to autoimmune and allergic disorders [11].

In this regard, it has been hypothesized that the GABAergic system may play a crucial role in the development of FS, suggesting a potential link between type 1 diabetes, autoimmune endocrine disorders, and FS through shared pathophysiological mechanisms [9]. According to Nataf et al., antibodies against GAD65, which are implicated in both type 1 diabetes and central nervous system disorders, often arise from two synergistic risk factors: immune challenges and psycho-emotional stress. This combination increases the likelihood of T cell priming against GAD65, potentially leading to both type 1 diabetes and central nervous system disorders [12]. Antibody spreading is a known mechanism in autoimmune diseases, and the propagation of GAD65 antibodies has been observed in individuals with type 1 diabetes, although antibody levels were not consistently high enough to predict autoreactivity in infiltrated tissues [13]. Proteomics could be employed to test this hypothesis regarding why type 1 diabetes (DM1) is more closely associated with FS than type 2 diabetes (DM2). Such research aims to identify immune-related proteins, including T lymphocyte markers and the presence of GAD65 or other superantigens, along with their corresponding antibodies. Nonetheless, disturbances in the GABAergic system could contribute to a fear of physical activity, and movement neglect has been identified as a significant risk factor for the development of FS [8].

A study by Kawahara et al. analyzed the proteomic profiles of FS in different regions of the shoulder capsule across various patient groups. Their analysis compared twelve patients with severe primary FS to seven patients with FS and rotator cuff tears, who served as a control group. Significant proteomic differences were found between the two groups, with several proteins highlighted for their clinical relevance [14]. Notably, proteins involved in the PI3K-Akt and PPAR signaling pathways—key components of insulin and leptin metabolism—were more prominent in patients with primary FS [15,16]. This increased signaling activity suggests the heightened involvement of insulin and leptin pathways in the pathophysiology of primary FS, potentially linked to insulin and leptin resistance [16,17]. Conversely, the proteome of patients with rotator cuff damage was more closely related to direct tissue injury rather than metabolic disturbances. These findings were supported by the detection of proteins indicating Staphylococcus aureus infection, antigen processing and presentation, and lysosome/phagosome activity [14]. The involvement of phagosomal and lysosomal activity suggests the presence of damage-associated molecular patterns (DAMPs) and pathogen-associated molecular patterns (PAMPs) [18,19]. In cases of FS following rotator cuff damage, barrier disruption between the blood and capsule may underlie symptomatology. This distinction implies that patients with primary FS may benefit from treatments targeting lifestyle modifications and metabolic interventions to address insulin and leptin resistance, while those with traumatic FS may require more orthopedic-focused approaches. The study also concluded that primary and secondary FS have distinct etiologies and pathophysiologies, necessitating differentiated therapeutic strategies [9,14].

Additionally, Hand et al. identified immune cell infiltration in the shoulder capsules of 22 patients with refractory primary FS. Mast cells were the most prevalent, followed by T lymphocytes, B lymphocytes, and macrophages, indicating a chronic inflammatory state in primary FS. The interaction between mast cells and fibroblasts suggests that inflammation drives fibrosis through mast cell-mediated mechanisms [20]. The presence of T and B lymphocytes, components of the adaptive immune system, raises the possibility of an autoimmune reaction targeting fibrotic tissue in FS, although alternative hypotheses may exist. Latest studies utilizing single-cell RNA sequencing (scRNA-seq) to map the cellular landscape of frozen shoulder tissue identified key cell types and their roles in the resolution of inflammatory fibrosis. The main findings suggest that FS differs from other fibrotic conditions because it is a self-limiting disease, with pro-inflammatory and fibrosis-resolving macrophages (MERTK+ macrophages) coexisting in the affected tissues. These macrophages interact with specific fibroblast subtypes (DKK3+ and POSTN+ fibroblasts) to promote matrix remodeling and inflammation resolution. Moreover, the study found similarities in the gene expression profiles of MERTK+ macrophages in FS and those in synovial tissues from rheumatoid arthritis patients in remission, supporting the hypothesis that these macrophages facilitate the resolution of inflammation. The study suggests that the cellular mechanisms for resolving FS fibrosis may be pre-established during human shoulder development. These findings open avenues for new therapeutic strategies aimed at promoting resolution of fibrosis in chronic inflammatory diseases by targeting the interactions between specific macrophage and fibroblast populations [21].

Despite growing evidence linking metabolic dysfunction with FS pathophysiology, the precise mechanisms remain unclear. The interplay between leptin resistance, JAK-STAT signaling, and immune responses has not been thoroughly investigated in FS, leading to a gap in understanding its systemic nature. This review aims to: (1) explore the link between metabolic dysregulation and immune dysfunction in FS, (2) examine the role of JAK-STAT signaling in chronic inflammation and fibrosis, (3) compare JAK-STAT signaling with other fibrotic pathways such as TGF-β, and (4) propose novel therapeutic strategies targeting metabolic–immune interactions in FS.

## 2. Scoping the Role of JAK-STAT Signaling and MERTK+ Macrophages in Inflammatory Fibrosis Resolution

The aforementioned discovery aligns with the hypothesis that metabolic signals, potentially mediated by leptin, influence the activity of these immune cells. Leptin’s role in energy regulation may extend to regulating the metabolic energy requirements of immune cells, particularly macrophages and fibroblasts, thereby influencing their capacity for fibrosis resolution. Given leptin’s dual role in metabolism and immunity, it is plausible that disruptions in leptin signaling—such as those observed in metabolic syndrome and obesity—contribute to prolonged inflammation and delayed fibrosis resolution in FS [22,23]. The cellular energy demands of MERTK+ macrophages and fibroblasts during matrix remodeling may be influenced by leptin sensitivity and overall metabolic health. Hypothetically, enhancing leptin sensitivity or targeting its downstream signaling pathways could improve the efficiency of these immune cells in resolving fibrosis, providing a potential therapeutic target. Thus, leptin may act as a bridge between metabolic dysfunction and impaired immune responses, perpetuating conditions like FS unless metabolic and immune homeostasis are restored [24]. The role of leptin and metabolic signals becomes even more pronounced when considering the JAK-STAT signaling pathway, as discussed in Sarapultsev et al. [25]. JAK-STAT is a key signaling mechanism in both immune and metabolic regulation, with direct implications for inflammation and cellular stress responses [26]. Dysregulation of this pathway, such as through leptin resistance, can perpetuate chronic inflammation and hinder the resolution of fibrosis, as seen in conditions like FS [10]. This pathway interacts with the immune system at various levels, promoting or dampening inflammatory responses based on metabolic inputs, such as leptin levels and signaling [27].

The presence of leptin resistance in FS patients may impair the proper functioning of the JAK-STAT pathway, leading to prolonged inflammation and delayed resolution of fibrosis [28]. This highlights the need for therapeutic interventions targeting both metabolic dysfunction and immune regulation, such as enhancing leptin sensitivity or directly modulating the JAK-STAT pathway to restore immune and metabolic balance in affected tissues [29]. Future research should focus on the combined role of metabolic regulators, like leptin, and immune-modulating pathways, like JAK-STAT, in promoting tissue repair and fibrosis resolution in frozen shoulder [9]. The JAK-STAT signaling pathway, a critical mediator of immune responses and leptin signaling, may also be implicated in FS pathophysiology. In the context of leptin resistance, dysregulation of the JAK-STAT pathway exacerbates inflammatory responses, activating transcription factors involved in fibrosis and tissue remodeling [30]. This suggests that therapies targeting JAK-STAT signaling could potentially mitigate the chronic inflammation and fibrosis characteristic of FS [31]. In this regard, a key aspect of fibrosis progression is the dysregulation of transcription factors, which are downstream targets of the JAK-STAT pathway [32]. Activation of STAT proteins, such as STAT3, has been implicated in promoting fibroblast proliferation and collagen production, exacerbating fibrotic responses [33]. The continuous stimulation of STAT3 in response to cytokines and growth factors, such as TGF-β, leads to unchecked ECM accumulation, a process that is central to the fibrotic pathology observed in FS [34]. Recent transcriptomic analyses from patients with FS reveal the upregulation of genes involved in immune responses and extracellular matrix modification, such as MMP9 and MMP13, which are closely associated with collagen degradation and fibrosis progression [35]. These findings suggest that metabolic dysregulation, mediated by leptin resistance, may directly influence the cellular processes that perpetuate inflammation and fibrosis in FS.

## 3. JAK-STAT, TGF-β, and Leptin Signaling in Frozen Shoulder

The TGF-β signaling pathway is well recognized as a central driver of fibrosis across multiple organ systems, including the musculoskeletal system. Elevated TGF-β activity has been implicated in fibroblast proliferation, extracellular matrix (ECM) deposition, and persistent myofibroblast activation, all of which contribute to joint capsule fibrosis and stiffness [36,37]. However, recent evidence suggests that the JAK-STAT pathway may also modulate fibroblast function and ECM production, establishing an important link between immune-mediated inflammation and fibrotic progression [38,39].

Notably, leptin signaling appears to intersect with both JAK-STAT and TGF-β pathways. Leptin promotes TGF-β-induced fibroblast differentiation into myofibroblasts in various fibrotic conditions, including pulmonary and hepatic fibrosis [40,41]. Moreover, studies suggest that STAT3 activation can directly upregulate TGF-β expression, amplifying fibrotic responses [42]. In contrast, leptin resistance may lead to a dysregulated JAK-STAT response, potentially altering its interaction with TGF-β and promoting a sustained fibrotic environment [43].

Both TGF-β and JAK-STAT pathways contribute to fibrosis progression, but through distinct and complementary mechanisms. TGF-β primarily drives fibrosis via SMAD-dependent pathways, promoting fibroblast-to-myofibroblast differentiation, ECM production, and tissue remodeling [36,37]. The JAK-STAT pathway, on the other hand, is primarily involved in immune regulation, but also plays a role in fibroblast activation and ECM remodeling, particularly through STAT3-mediated transcriptional regulation of fibrotic genes [38,39]. The crosstalk between these pathways is evident in various fibrotic conditions, where persistent STAT3 activation has been linked to increased TGF-β expression, reinforcing the fibrotic response [42].

The interplay between leptin resistance, JAK-STAT signaling, and TGF-β in FS remains an area of significant interest. Leptin resistance disrupts homeostatic metabolic signaling, which in turn influences immune and fibrotic pathways, creating a chronic inflammatory state that favors fibrosis progression. The effects of leptin on TGF-β signaling could be direct, through its influence on fibroblast activation, or indirect, by modulating the inflammatory microenvironment that sustains TGF-β activity [40,41]. Given that leptin resistance is closely linked to metabolic dysfunction, obesity, and chronic inflammation, its impact on both JAK-STAT and TGF-β could provide a unifying explanation for the metabolic–immune dysregulation observed in FS [43].

While JAK-STAT and TGF-β function as independent pathways, their interaction in the fibrotic progression of FS remains underexplored. Given the evidence that leptin influences both pathways, further research is warranted to determine whether leptin resistance disrupts this balance and fosters a chronic inflammatory-fibrotic state in FS. Targeting both pathways simultaneously—through JAK-STAT inhibitors, TGF-β modulators, or metabolic interventions aimed at improving leptin sensitivity—could provide a novel therapeutic strategy for FS patients.

## 4. Chronic Neuroinflammation, Leptin Resistance, and JAK-STAT Signaling

The coexistence of both pro-inflammatory and fibrosis-resolving macrophages (MERTK+) within the fibrotic tissues of frozen shoulder (FS) may be further explained through the lens of neuroinflammation and leptin resistance, particularly regarding the JAK-STAT signaling pathway [21]. Neuroinflammation, a state of chronic inflammation within the central nervous system (CNS), often results from a dysregulated immune response, driven by the JAK-STAT pathway [44]. This pathway is activated by various cytokines that promote both immune and inflammatory responses, playing a crucial role in diseases such as multiple sclerosis and Parkinson’s disease [34].

Leptin resistance at the hypothalamic level, which is often present in individuals with metabolic syndrome, can exacerbate neuroinflammation by disrupting the normal regulation of energy balance and immune responses [45]. Leptin normally acts on hypothalamic receptors to regulate appetite and energy expenditure, but when leptin signaling is impaired, a state of chronic low-grade inflammation ensues [46]. This inflammatory state may spill over into peripheral tissues, such as those involved in FS, creating an environment where pro-inflammatory and fibrosis-resolving macrophages coexist [47]. The JAK-STAT pathway plays a central role in both the central and peripheral inflammatory processes, amplifying the inflammatory signals in response to neuroinflammation [34,48].

In FS, this disrupted signaling could manifest as a failure to effectively transition macrophages from a pro-inflammatory to a pro-resolving phenotype, leading to the persistence of fibrosis [38,49]. The involvement of JAK-STAT signaling in neuroinflammation suggests that targeting this pathway could mitigate not only the peripheral fibrotic response, but also the central neuroinflammatory drivers of FS [26,50]. The pathway’s role in activating transcription factors associated with fibrosis and inflammation further underscores its importance [51,52]. Future research should explore the potential cross-talk between neuroinflammation, JAK-STAT signaling, and leptin resistance in FS, as this might unveil novel therapeutic strategies aimed at resolving both neuroinflammation and peripheral fibrosis [47]. Furthermore, in FS, the role of leptin may be further complicated by the inhibition of its signaling by SOCS3, which binds to the leptin receptor, particularly at sites necessary for STAT3 activation [53]. This attenuation of leptin signaling may exacerbate both peripheral inflammation and fibrosis because it disrupts the balance of macrophage activation [54]. SOCS3, by inhibiting the JAK-STAT signaling pathway, prevents effective immune resolution and promotes sustained inflammation. Moreover, SOCS3’s role extends beyond leptin resistance [55]. It is also implicated in insulin resistance, which contributes to the broader metabolic dysregulation observed in FS patients [7]. In these individuals, overexpression of SOCS3 in both hypothalamic and peripheral tissues reduces the effectiveness of insulin signaling, further fueling systemic inflammation [54]. The link between metabolic disorders and fibrotic diseases, like FS, may thus be mediated, in part, by the SOCS3-induced blockade of both leptin and insulin pathways, perpetuating chronic inflammation and tissue fibrosis.

This insight prompts further investigation into the metabolic regulation of immune cell functions in FS and other chronic fibrotic diseases. It suggests that therapies aimed at improving metabolic health—particularly those enhancing leptin sensitivity—could also facilitate immune resolution processes, opening new avenues for treating FS by targeting both the metabolic and immune aspects of the disease.

**Figure 1:**
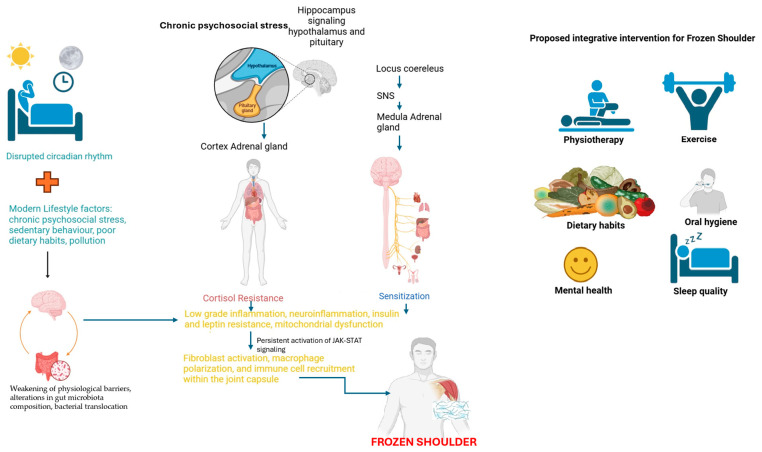
Note. Pathophysiological mechanisms and integrative treatment approaches in frozen shoulder. This figure provides a comprehensive overview of the multifactorial pathophysiology of frozen shoulder (FS) and the proposed integrative treatment strategies. On the left side of the figure, key lifestyle and environmental factors, such as circadian rhythm desynchronization and modern lifestyle influences (chronic psychosocial stress, sedentary behavior, poor dietary habits, and pollution), are depicted as major contributors to systemic dysregulation. When sustained over time, these factors promote low-grade inflammation, neuroinflammation, and a state of insulin and leptin resistance, disrupting normal metabolic and immune homeostasis. This condition fosters the weakening of physiological barriers, alterations in gut microbiota composition, and bacterial translocation, further amplifying systemic inflammation and immune dysregulation. In the central portion of the figure, the interconnected pathways of metabolic, immune, and neuroendocrine dysfunctions are highlighted. Chronic psycho-emotional stress and circadian rhythm disruption lead to persistent activation of the sympathetic nervous system (SNS) and the hypothalamic–pituitary–adrenal (HPA) axis, which, over time, results in cortisol resistance and SNS sensitization. This prolonged neuroendocrine dysfunction fosters low-grade inflammation and neuroinflammation, further impairing insulin and leptin signaling. Leptin resistance, in particular, contributes to a sustained activation of the JAK-STAT signaling pathway, which is known to promote chronic inflammation and fibrotic processes. The dysregulation of this pathway is likely responsible for the coexistence of pro-inflammatory and fibrosis-resolving macrophages in FS, perpetuating a pathological state where fibrosis is initiated but not efficiently resolved. Furthermore, gut microbiota dysbiosis and bacterial translocation contribute to the systemic immune activation, exacerbating the inflammatory environment and fibrosis progression in FS. On the right side of the figure, a multi-targeted, integrative treatment strategy for FS is proposed, addressing both systemic metabolic–immune imbalances and localized tissue pathology. These interventions include physiotherapy, exercise, dietary habits, oral health, mental health, and sleep quality.

The role of chronic psychosocial stress in FS pathophysiology is an emerging area of interest, particularly in its interaction with metabolic and immune dysregulation. Psychological factors such as pain-related fear, anxiety, and depression have been identified as key prognostic markers in FS, influencing functional disability, pain intensity, and recovery duration [56]. Stress-induced activation of the hypothalamic–pituitary–adrenal (HPA) axis and the resultant low-grade systemic inflammation may create a metabolic and immunological context that predisposes individuals to FS [9]. Chronic exposure to stressors not only contributes to increased circulating levels of pro-inflammatory cytokines, but also disrupts the homeostatic regulation of leptin and insulin signaling, further exacerbating immune dysregulation and fibrotic remodeling in FS. In this context, leptin resistance may serve as a metabolic link between chronic psychosocial stress and the inflammatory processes driving FS, as persistent stress is known to impair leptin signaling, fostering behavioral patterns characterized by reduced physical activity and heightened pain perception [8]. These findings suggest that targeting stress-related metabolic dysregulation, alongside immune and fibrotic pathways, could offer a more integrative therapeutic approach to FS management. Future studies should explore the potential of interventions aimed at mitigating stress-induced immune activation and leptin resistance as a novel strategy to improve both clinical outcomes and patient quality of life in FS (See Figure 1).

## 5. Low-Grade Infections, Altered Microbiota, and Leptin Resistance

Interestingly, systemic leptin levels are known to decrease in states of malnutrition and starvation, which underscores leptin’s role as a mediator between nutritional status and immune function [57]. Leptin deficiency is linked to heightened vulnerability to various infections [58]. Furthermore, certain infections can mimic malnutrition by downregulating leptin levels, contributing to an immune-compromised state [59]. For example, a significant drop in leptin levels during starvation has been associated with increased sensitivity to endotoxins like lipopolysaccharides (LPS) and TNF-α in animal models. However, leptin replacement has been shown to reverse these effects, protecting against fasting-induced immune deficiencies, such as lymphopenia [60,61].

Phagocytosis, a key immune mechanism for eliminating pathogens, is enhanced by leptin, which stimulates macrophage activity and prevents apoptosis in multiple immune cells involved in both innate and adaptive immunity [62]. Numerous studies support the idea that leptin supplementation can reduce infections caused by pathogens such as *Listeria monocytogenes*, *Klebsiella pneumoniae*, *Escherichia coli*, and *Mycobacterium tuberculosis* by boosting macrophage phagocytosis [10,59,63]. Leptin also plays a crucial role in combating sepsis, a severe inflammatory response that can lead to organ failure and death. In leptin-deficient mice, exogenous leptin administration improved survival rates during sepsis by reducing systemic IL-6 levels and controlling inflammation [10,64]. In humans, higher leptin levels have been observed in survivors of sepsis compared to non-survivors, underscoring leptin’s protective role in immune responses during infection [59].

Obesity, on the other hand, is associated with elevated leptin levels, which can promote chronic low-grade inflammation by activating both innate and adaptive immune cells [65]. This pro-inflammatory environment contributes to the breakdown of immune tolerance, priming immune cells for a Th1-dominated response [10,66]. This persistent inflammation heightens the risk of developing metabolic diseases such as cardiovascular disease and type 2 diabetes, as well as autoimmune conditions like multiple sclerosis, thyroiditis, rheumatoid arthritis, and inflammatory bowel disease [67].

Chronic inflammation, whether due to infection or autoimmune disease, can lead to leptin resistance, particularly in the hypothalamus [68]. This leptin resistance impairs appetite regulation and energy expenditure, further exacerbating obesity [69]. In obesity, the interaction between hypertrophic adipocytes and immune cells, such as macrophages, leads to an overproduction of pro-inflammatory cytokines and adipokines, like leptin [70]. These molecules perpetuate local and systemic inflammation, contributing to leptin resistance at both the peripheral and central levels [71]. Inflammatory cytokines activate signaling pathways, such as NF-κB, which induce suppressors of cytokine signaling (SOCS3) and protein tyrosine phosphatases (PTP1B), further inhibiting leptin receptor signaling via the JAK/STAT pathway [26,72,73].

Emerging research highlights the role of gut microbiota in regulating systemic metabolism, particularly energy balance, glucose homeostasis, and low-grade inflammation associated with obesity [74,75,76]. Alterations in the gut microbiota, such as those caused by a high-fat diet, have been linked to a reduction in beneficial bacterial populations, like *Bifidobacterium* spp., *Lactobacillus* spp., and *Roseburia* spp. [77,78,79]. The interaction between gut microbiota and the immune system is mediated by pattern recognition receptors like Toll-like receptors (TLRs), which detect microbial components such as LPS. Fatty acids can stimulate innate immunity by interacting with the TLR4/CD14 complex, further promoting inflammatory responses [80]. Alterations in the gut microbiota have been implicated in the development of obesity and its related metabolic disorders, with evidence suggesting that modulating the gut microbiota can influence leptin sensitivity and metabolic outcomes [81,82,83].

Interestingly, the gut microbiota also appears to regulate leptin action, with studies showing that dietary interventions, like prebiotics, can improve leptin sensitivity in obese and diabetic mice [84]. This suggests that gut microbiota modulation may offer a novel therapeutic strategy for restoring leptin sensitivity and addressing metabolic dysregulation in FS.

Additionally, recent findings suggest a possible infectious etiology in frozen shoulder (FS), linked to bacteria that commonly inhabit human skin, such as *Propionibacterium acnes* (*P. acnes*) [85,86,87]. Although some studies argue against this hypothesis, others have found evidence of infection in FS patients. For instance, markers such as HMGB1, IL-33, S100A8, and S100A9 were elevated in the joint capsules of FS patients, supporting the notion of a low-grade infection contributing to the pathology [88]. *P. acnes*, often dismissed as a contaminant due to its slow growth in anaerobic conditions, has been implicated in post-surgical infections and other conditions, like disk herniation [89,90,91]. The systemic spread of these bacteria, along with species like *Streptococcus epidermis* and *Corynebacterium propinquum*, could contribute to persistent low-grade infections that play a role in the chronic inflammation observed in FS [92].

Bacterial infiltration can occur through daily activities, such as tooth brushing, leading to microbial dissemination via the bloodstream [93]. These bacteria may enter tissues with low oxygen levels, such as the intervertebral disks and possibly the shoulder joint, particularly in areas with reduced movement [8,94]. Such low-grade infections may be an under-recognized factor in the development of FS and other inflammatory conditions [95].

## 6. Discussion

This review highlights the complex interplay between leptin resistance, JAK-STAT signaling, metabolic dysregulation, and immune dysfunction in the pathophysiology of frozen shoulder (FS). We summarize evidence suggesting that FS shares common inflammatory and fibrotic pathways with metabolic disorders, particularly in conditions characterized by chronic low-grade inflammation, insulin resistance, and dysregulated adipokine signaling. Leptin, traditionally recognized for its role in energy homeostasis, emerges as a key immunometabolic regulator, influencing fibroblast activation, macrophage polarization, and immune cell recruitment within the joint capsule. Moreover, we discuss how JAK-STAT dysregulation, particularly through leptin resistance and SOCS3 overexpression, may perpetuate chronic inflammation and fibrosis in FS, similar to its role in other fibrotic conditions. Recent findings demonstrate the coexistence of both pro-inflammatory and fibrosis-resolving macrophages (MERTK+) in FS tissue, suggesting that metabolic and immune cross-talk influences the progression and resolution of fibrosis, a concept that aligns with recent transcriptomic and proteomic studies. Additionally, the review explores the potential role of chronic psychosocial stress as a driver of immune–metabolic dysregulation and discusses emerging evidence linking gut microbiota alterations and bacterial translocation to systemic inflammation and immune priming in FS. These insights broaden the traditional understanding of FS as a purely orthopedic condition, instead positioning it as a multisystem disorder influenced by metabolic, inflammatory, and immune dysfunctions.

A recent study reveals the coexistence of both pro-inflammatory macrophages and fibrosis-resolving MERTK+ macrophages within the same affected tissue in FS, raising important questions regarding the underlying metabolic and signaling mechanisms that allow such cellular dynamics [21]. In traditional models of inflammation, pro-inflammatory macrophages (often referred to as M1) dominate the early stages of the immune response, followed by a transition to pro-resolving macrophages (M2) as the inflammation subsides [96]. However, the coexistence of these two distinct macrophage phenotypes in the fibrotic tissue of FS suggests a more complex regulatory environment, which is potentially driven by metabolic and immunological factors [97].

One possible explanation for this paradox may lie in the metabolic state of the tissue, particularly in relation to leptin resistance [98]. Leptin, a key regulator of both metabolism and immune responses, plays a crucial role in macrophage polarization [99]. In the context of leptin resistance, which is commonly observed in metabolic syndromes such as obesity and type 2 diabetes, macrophages may exhibit a dysregulated response [98]. This dysregulation could impair the typical transition from pro-inflammatory to pro-resolving states, allowing both phenotypes to coexist within the same tissue [100]. Specifically, leptin resistance may impair the energetic capacity of macrophages, altering their metabolic flexibility, which is required for the shift toward a fibrosis-resolving phenotype [101]. The inability to efficiently switch from glycolysis (which favors M1 polarization) to oxidative phosphorylation (which supports M2 activity) could result in the persistence of both pro-inflammatory and pro-resolving macrophages in the fibrotic environment of FS [102].

Moreover, the JAK-STAT signaling pathway, which is critically involved in both immune regulation and leptin signaling, may further contribute to this phenomenon [103]. Dysregulation of the JAK-STAT pathway due to leptin resistance can exacerbate chronic inflammation by promoting the persistence of inflammatory macrophages while simultaneously activating downstream signals that encourage tissue remodeling and fibrosis resolution [27]. In the presence of leptin resistance, the failure to adequately suppress the pro-inflammatory arm of the JAK-STAT pathway may lead to an environment where fibrosis-resolving processes are initiated but not fully effective, thereby allowing these seemingly opposing macrophage populations to coexist [51].

Thus, the coexistence of pro-inflammatory and fibrosis-resolving macrophages in FS tissue may be indicative of a metabolic and signaling imbalance, where leptin resistance and JAK-STAT pathway dysregulation prevent the efficient resolution of inflammation while simultaneously promoting fibrosis. This dynamic highlights the need for further research into how metabolic interventions, particularly those targeting leptin sensitivity and JAK-STAT modulation, could restore proper immune balance and improve fibrosis outcomes in FS.

## 7. Current Treatments and Future Directions

The treatment of FS remains a challenging and complex endeavor due to the multifactorial nature of the disease, involving both metabolic and inflammatory components. Current therapeutic approaches range from non-surgical interventions, such as physical therapy, corticosteroid injections, and manual mobilization, to more invasive procedures, like arthroscopic capsular release and hydrodilation [3]. However, these methods primarily focus on symptom relief and improving shoulder mobility, with limited effectiveness in addressing the underlying inflammatory and fibrotic processes [104].

### Conventional Therapies and Their Limitations

Non-surgical approaches, such as corticosteroid injections, have demonstrated effectiveness in reducing pain and improving joint function during the early “freezing” phase of FS. Nonetheless, the long-term impact of corticosteroids remains limited, as they do not target the underlying causes of fibrosis and inflammation [3,104]. Similarly, physical therapy and manual mobilization are beneficial for improving the range of motion, particularly in the later stages of FS, but they also fall short in addressing the root metabolic and inflammatory factors driving disease progression [8,105].

Among non-surgical treatments, corticosteroid injections are widely used to reduce pain and improve joint function during the early “freezing” phase of FS [106]. Despite their short-term efficacy, long-term corticosteroid use does not modify the disease course and is associated with significant risks, including joint deterioration, cartilage degeneration, and systemic metabolic side effects, such as hyperglycemia and insulin resistance, particularly in patients with pre-existing metabolic disorders [107]. Similarly, physical therapy and manual mobilization remain the mainstay of conservative treatment, with benefits in the range of motion improvement, particularly in the later stages of FS [108,109]. However, overly aggressive mobilization may worsen pain and inflammation, potentially prolonging recovery [110].

For patients with persistent symptoms, surgical interventions such as arthroscopic capsular release and hydrodilation are considered. While capsular release is effective in improving mobility, it carries risks such as postoperative stiffness, adhesion reformation, and persistent pain [111]. Hydrodilation, which involves the injection of large fluid volumes into the joint capsule to increase capsular distension, has shown variable success rates and does not target the fibrotic and metabolic dysfunction underlying FS [112].

## 8. Emerging Therapeutic Approaches

Recent insights into metabolic dysregulation, particularly leptin resistance, suggest that FS should not only be viewed as a mechanical disorder, but also as a metabolic–immunological condition. Given the role of leptin in immune regulation and fibrosis, novel therapeutic strategies targeting leptin sensitivity, JAK-STAT signaling, and microbiome modulation have gained attention [8].

### 8.1. Targeting Leptin Resistance and Metabolic Dysregulation

Given the potential role leptin may play in FS pathophysiology, therapeutic interventions aimed at improving leptin sensitivity could prove beneficial. Studies have shown that exercise, particularly resistance and aerobic training, can improve leptin sensitivity and reduce systemic inflammation [8,105]. In addition, dietary modifications, such as time-restricted feeding or intermittent fasting, have been found to modulate leptin levels, offering a potential avenue for metabolic correction in FS patients [105]. However, while restoring leptin sensitivity could theoretically ameliorate fibrosis and inflammation, potential risks remain. Excessive leptin modulation could impact systemic metabolism, potentially influencing appetite regulation, insulin sensitivity, and immune function [113]. Further research is required to determine the optimal balance between therapeutic benefits and metabolic stability in FS treatment.

### 8.2. JAK-STAT Pathway as a Target

Persistent activation of this pathway contributes to the overproduction of pro-inflammatory cytokines, macrophage polarization, and fibroblast activation, leading to excessive extracellular matrix deposition and fibrosis [34,48]. Thus, the JAK-STAT signaling pathway, activated by inflammatory cytokines and impaired leptin signaling, may play a crucial role in both immune regulation and fibrotic progression in FS. Therapies aimed at modulating the JAK-STAT pathway could reduce inflammation and promote tissue repair, making it a promising target for FS treatment [8,105]. However, JAK-STAT inhibitors, which are already in clinical use for autoimmune and inflammatory conditions like rheumatoid arthritis, have shown promise in reducing fibrosis by modulating macrophage activity and fibroblast proliferation [114]. Moreover, their immunosuppressive effects pose significant risks, including increased susceptibility to infections, impaired immune surveillance, and potential long-term metabolic alterations [115]. Future research should aim to determine whether selective JAK-STAT modulation can provide anti-fibrotic benefits while minimizing immune suppression, potentially making it a viable therapeutic option for FS.

### 8.3. Gut Microbiota Modulation 

Recent studies have highlighted the role of the gut microbiota in regulating systemic inflammation and metabolic health, including leptin sensitivity [116]. Alterations in gut microbiota composition, often observed in obesity and metabolic syndrome, can exacerbate leptin resistance and inflammatory processes via bacterial translocation and LPS-mediated activation of the JAK-STAT pathway [117]. Probiotic and prebiotic interventions may offer a novel therapeutic strategy to improve metabolic and immune regulation in FS patients, potentially reducing the chronic inflammation and fibrosis associated with the condition [8,118]. However, the precise role of microbiota-driven inflammation in FS remains to be fully elucidated, warranting further clinical investigation.

## 9. Future Research Directions

Given the complex interplay of metabolic, inflammatory, and fibrotic processes in FS, future research must explore novel pathways that may provide insight into the underlying mechanisms of this condition. One promising area of investigation involves the relationship between mental health, mitochondrial dysfunction, circadian rhythm disruption, leptin resistance, and the JAK-STAT signaling pathway, which together could play a pivotal role in FS progression.

### 9.1. Lifestyle Interventions as a Potential Therapeutic Approach in FS

In light of the growing evidence linking chronic psychosocial stress, metabolic dysregulation, and immune dysfunction in FS, lifestyle interventions emerge as a promising avenue for disease management [24]. Modern lifestyle factors, including sedentary behavior, poor dietary habits, and chronic exposure to psychosocial stressors, have been implicated in the disruption of homeostatic regulatory systems, including the hypothalamic–pituitary–adrenal (HPA) axis and the autonomic nervous system [24]. These disruptions, in turn, contribute to chronic low-grade inflammation, gut barrier dysfunction, and neuroimmune activation, which have been proposed as key drivers of immune-mediated musculoskeletal conditions such as FS [119]. From a psychoneuroimmunological perspective, interventions that promote physical activity, circadian alignment, and metabolic flexibility could mitigate the sustained immune activation and metabolic disturbances associated with shoulder pain [120]. Regular exercise has been shown to improve leptin and insulin sensitivity, while also exerting anti-inflammatory effects through modulation of the gut microbiome and systemic cytokine profiles [121]. Additionally, circadian rhythm regulation through light exposure, sleep hygiene, and structured feeding patterns has been proposed as an essential component in restoring immune–metabolic balance, particularly in individuals experiencing stress-related immune dysfunction [122]. Given the interplay between environmental stressors, immune dysregulation, and FS progression, future research should focus on integrative lifestyle-based interventions that address both the physiological and psychosocial dimensions of the disease. These approaches may hold significant potential in improving patient outcomes while reducing reliance on pharmacological therapies that primarily target symptom relief rather than the underlying pathophysiology of FS.

### 9.2. Mitochondrial Dysfunction and FS Progression

Mitochondrial health is critical for maintaining cellular energy homeostasis and regulating immune responses. Mitochondrial dysfunction, often a result of chronic inflammation and metabolic stress, is associated with decreased ATP production, increased oxidative stress, and impaired tissue repair [123]. In the context of FS, mitochondrial dysfunction may exacerbate the chronic inflammation and fibrosis observed in the joint capsule [8]. As immune cells, such as macrophages, rely on mitochondrial energy production to facilitate their transition from pro-inflammatory (M1) to fibrosis-resolving (M2 or MERTK+), mitochondrial dysfunction could delay this transition, prolonging inflammation and fibrosis [21]. Understanding how mitochondrial impairment influences macrophage function and fibroblast activity in FS could provide valuable insight into the disease’s metabolic underpinnings and suggest potential therapeutic targets aimed at restoring mitochondrial function to improve tissue repair.

### 9.3. Disruption of Circadian Rhythms

Circadian rhythms regulate a wide array of physiological processes, including immune function and energy metabolism. Disruption of these rhythms, whether due to sleep disorders, shift work, or other environmental factors, has been linked to metabolic diseases and chronic inflammation [124]. In shoulder disorders, the dysregulation of circadian rhythms could further impair the already-compromised metabolic pathways, such as leptin signaling, thereby perpetuating the disease state [120]. Leptin levels are known to follow a circadian pattern, and disruptions in these rhythms may worsen leptin resistance, impairing the body’s ability to regulate energy balance and immune responses effectively [125,126]. Investigating how circadian rhythm disturbances contribute to metabolic dysregulation and inflammation in FS could open new avenues for chronotherapy-based interventions that realign circadian patterns to improve metabolic outcomes and reduce inflammation [127,128].

### 9.4. Leptin Resistance and JAK-STAT Pathway

Leptin resistance may play a central role in the chronic inflammation and metabolic disturbances observed in FS [24,129]. The JAK-STAT pathway, activated by inflammatory cytokines and impaired leptin signaling, is a critical mediator of immune responses and fibrosis [38]. In FS, persistent activation of the JAK-STAT pathway due to leptin resistance may promote the sustained activation of pro-inflammatory macrophages and fibroblasts, leading to excessive extracellular matrix deposition and tissue fibrosis [38]. Future research should focus on the precise mechanisms by which leptin resistance amplifies JAK-STAT signaling and how this contributes to the maintenance of a chronic inflammatory state in FS [129]. Targeting the JAK-STAT pathway in conjunction with restoring leptin sensitivity may provide a dual approach to reducing inflammation and fibrosis in FS patients.

## 10. Conclusions

This review highlights the potential critical role of metabolic–immune dysregulation, particularly leptin resistance and JAK-STAT signaling, in the pathophysiology of FS. The persistence of both pro-inflammatory and fibrosis-resolving (MERTK+) macrophages within FS tissue suggests a disrupted immune resolution process, potentially driven by metabolic impairments. Leptin resistance, a hallmark of metabolic disorders, not only contributes to sustained inflammation, but also disrupts macrophage polarization, delaying fibrosis resolution. This metabolic dysfunction is further amplified by chronic neuroinflammation, mitochondrial dysfunction, and circadian rhythm disruption, which collectively impair the transition from acute to resolving phases of inflammation.

The JAK-STAT pathway, as a central mediator of immune and metabolic responses, likely plays a pivotal role in maintaining this dysfunctional environment.

Given the complexity of FS pathogenesis, effective therapeutic strategies should move beyond symptom management and target the root causes of metabolic and immune dysregulation. Restoring leptin sensitivity, modulating JAK-STAT signaling, improving mitochondrial function, and aligning circadian rhythms emerge as promising avenues for intervention. Additionally, integrative approaches, including exercise, dietary modifications, gut microbiota-targeted therapies, and psychological stress management, may enhance immune resilience and metabolic homeostasis.

Future research should prioritize investigating the mechanistic links between metabolic dysfunction, immune dysregulation, and fibrosis progression in FS. A deeper understanding of how chronic stress, neuroendocrine alterations, and systemic inflammation intersect in FS pathophysiology may open new therapeutic opportunities, not only for FS, but also for other chronic inflammatory and fibrotic conditions. Addressing these interconnected mechanisms through precision medicine and personalized metabolic–immune interventions could transform FS management, offering long-term solutions that go beyond conventional orthopedic treatments.
